# Clinical and Functional Outcomes Following Intra-articular Platelet-Rich Plasma Injection for Knee Osteoarthritis: A Prospective Cohort Study

**DOI:** 10.7759/cureus.95297

**Published:** 2025-10-24

**Authors:** Thivagar Murugesan, Mahesh Mohankumar, Pedapati SSK Vijaya Guna Surya

**Affiliations:** 1 Trauma and Orthopaedics, Shrewsbury and Telford NHS Foundation Trust, Shrewsbury, GBR; 2 Trauma and Orthopaedics, Tagore Medical College, Chennai, IND; 3 Trauma and Orthopaedics, Sai Spoorthi Hospital, Chintalupadi, IND

**Keywords:** intra-articular therapy, knee osteoarthritis, prp injection, regenerative medicine, vas, womac-crd

## Abstract

Background and objective

Knee osteoarthritis (OA), a common degenerative joint disease, often leads to chronic pain and disability, particularly in middle-aged individuals. With growing interest in regenerative therapies, platelet-rich plasma (PRP) has emerged as a potential biological treatment due to its anti-inflammatory and reparative properties. This study aimed to evaluate the clinical and functional outcomes following intra-articular PRP injections in patients with early-stage knee OA.

Methodology

A prospective clinical trial was conducted at a tertiary care center involving 113 patients diagnosed with Kellgren-Lawrence grade 1 or 2 knee osteoarthritis. Each participant received a single intra-articular PRP injection and was followed for 24 weeks. Pain and functional outcomes were assessed using the visual analog scale (VAS) and the Western Ontario and McMaster Universities Osteoarthritis Index (WOMAC)-CRD at baseline, and at six, 12, and 24 weeks post-injection.

Results

At every follow-up, both VAS and WOMAC scores demonstrated significant reductions (p < 0.001). Patients with grade 1 OA showed greater improvement compared to those with grade 2 at all time points. While VAS scores decreased from 5.87 to 2.85, the average WOMAC score fell from 32.81 at baseline to 24.89 after 24 weeks. Better results were strongly connected with younger age and lower radiographic grade.

Conclusions

Our results align with existing research supporting the effectiveness of PRP in managing early osteoarthritis. The treatment showed consistent symptom relief, particularly in younger patients and those with lower radiographic grades. Over a six-month period, intra-articular PRP injections offered significant pain reduction and functional improvement in early-stage knee OA, establishing it as a safe and effective therapy.

## Introduction

Knee osteoarthritis (OA) is a common, progressive degenerative joint disease, especially prevalent in the elderly, that leads to significant pain, functional limitations, and a reduced quality of life. Knee OA is characterized by increased osteophyte formation, synovial inflammation, subchondral bone remodeling, and degeneration of articular cartilage, imposing a significant socioeconomic burden on healthcare systems worldwide [1,2]. Therapeutic choices for knee OA are mostly palliative despite the high prevalence and increasing incidence driven by aging populations and rising obesity rates. The primary goals are to alleviate symptoms, maintain joint mobility, and delay disease progression. While conventional therapies, such as nonsteroidal anti-inflammatory drugs (NSAIDs), corticosteroid injections, physical therapy, and lifestyle modifications, can offer temporary symptom relief, they often fail to halt the underlying degenerative process. Additionally, adverse effects and diminishing efficacy frequently limit their long-term use [3,4].

In recent years, regenerative medicine has emerged as a promising approach for managing musculoskeletal diseases, especially in the early and moderate stages of OA. Among these therapies, autologous platelet-rich plasma (PRP) treatment has gained considerable attention due to its potential to harness the body’s natural healing abilities [5]. Derived from the patient's own blood, PRP is a concentrated suspension of platelets in a small volume of plasma. When activated, these platelets release numerous bioactive molecules and growth factors - such as platelet-derived growth factor (PDGF), transforming growth factor-beta (TGF-β), vascular endothelial growth factor (VEGF), and insulin-like growth factor-1 (IGF-1) - all of which play key roles in tissue repair, inflammation regulation, angiogenesis, and maintaining cartilage health. Beyond the clinical alleviation offered by traditional intra-articular treatments, our biochemical findings suggest that PRP may have a potential disease-modifying effect [6,7]

Though the data supporting the efficacy of PRP in knee OA is mounting, significant variation still exists in preparation methods, platelet concentration, injection techniques, frequency, and result evaluation tools. Additionally, inconsistent results are influenced by differences in patient selection and OA severity across studies [8]. Although many randomized controlled trials and meta-analyses have shown PRP to be effective in reducing pain and function in knee OA relative to placebo, hyaluronic acid, or corticosteroids, there remains a need for robust prospective clinical data focusing on standardized protocols and clinically relevant outcome measures [9,10].

This prospective clinical trial aims to assess the clinical and functional results of intra-articular PRP injection in knee OA patients. The study seeks to offer quality evidence on the therapeutic efficiency of PRP in real-world clinical settings by using validated clinical scoring systems, such as the Western Ontario and McMaster Universities Osteoarthritis Index (WOMAC)-CRD (free Indian version) and the visual analog scale (VAS), and by following standardized preparation and administration protocols. Additionally, it aims to correlate functional improvements with symptom relief over a defined follow-up period, thereby contributing to the growing body of research on regenerative treatments for knee OA. We hope the findings will help guide future studies and clinical practice toward optimizing patient outcomes through biologically based therapies.

## Materials and methods

**Figure 1:**
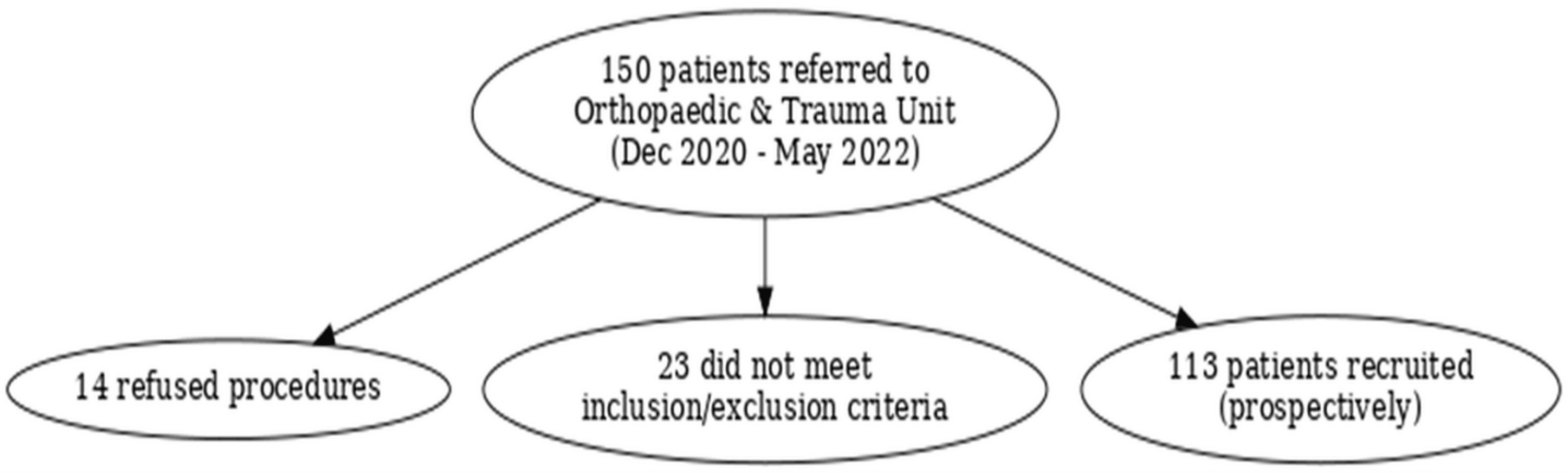
Flowchart for patient recruitment in the study on knee

The study prospectively evaluated patients from the Orthopaedic Department at General Hospital, Jayanagar, Bengaluru, between December 2020 and May 2022. Of the 150 participants initially enrolled, 14 declined to participate, and 23 were excluded, resulting in 113 patients being included in the study. The study included patients aged 40 years and above who had Kellgren-Lawrence grade 1 or 2 osteoarthritis and experienced chronic knee pain lasting more than three months and whose symptoms were not adequately managed with opioids and non-steroidal anti-inflammatory drugs (NSAIDs). Patients with immunosuppression, secondary OA, connective tissue disorders, malignancy, inflammatory knee joint diseases, or anticoagulant or antiplatelet medication within 10 days after injection were excluded. Additionally, individuals who received systemic corticosteroids within two weeks after PRP treatment were deemed ineligible. Figure 1 presents a flowchart depicting patient recruitment for the study.

Initial screening involved collaboration with a radiologist through clinical and radiological evaluations, including Kellgren and Lawrence grading. Eligible participants provided demographic details such as age, gender, and affected side using a standardized proforma and gave informed consent. Necessary blood tests were performed before proceeding with the intervention

Each participant provided 20 ml of venous blood to prepare autologous PRP, which was promptly transferred into ACD tubes. The blood underwent two rounds of centrifugation: first at 1600 rpm for 15 minutes to separate the erythrocyte-rich red layer, leukocyte-rich white layer, and the platelet- and growth factor-rich yellow plasma layer. The plasma layer was then centrifuged again at 2800 rpm for seven minutes to concentrate the platelets. Platelet-poor plasma was discarded, while the PRP was retained with a platelet concentration ranging from 1,000,000 to 15,000,000 platelets per microliter. After preparing the skin with povidone-iodine, 3-5 ml of PRP was injected aseptically into the knee joint using a 22-gauge needle via the anteromedial or anterolateral approach. Patients were advised to perform active range-of-motion exercises post-injection to aid PRP distribution.

Clinical and functional outcomes were evaluated using the VAS for pain and the WOMAC-CRD (free Indian version) for osteoarthritis. The VAS, a 0-10 scale, assessed pain intensity at baseline and at six, 12, and 24 weeks after the injection. The WOMAC-CRD scores, which measure pain, stiffness, and physical function, were also recorded. All participants were followed up at six, 12, and 24 weeks post-intervention to track progress and document any adverse events [11]

Data were entered using Microsoft Excel and analyzed with SPSS Statistics version 20 (IBM Corp., Armonk, NY). Descriptive statistics, including means, percentages, and standard deviations (SD), were calculated. With a significance threshold set at p < 0.05, paired t-tests and independent t-tests were employed to compare scores before and after the intervention.

The Institutional Ethics Committee of General Hospital, Jayanagar, granted ethical clearance, and the trial registration number is GHJ/DNB/01/2022-23; all subjects provided signed informed consent. The confidentiality and privacy of participants were maintained throughout the study.

## Results

**Figure 2:**
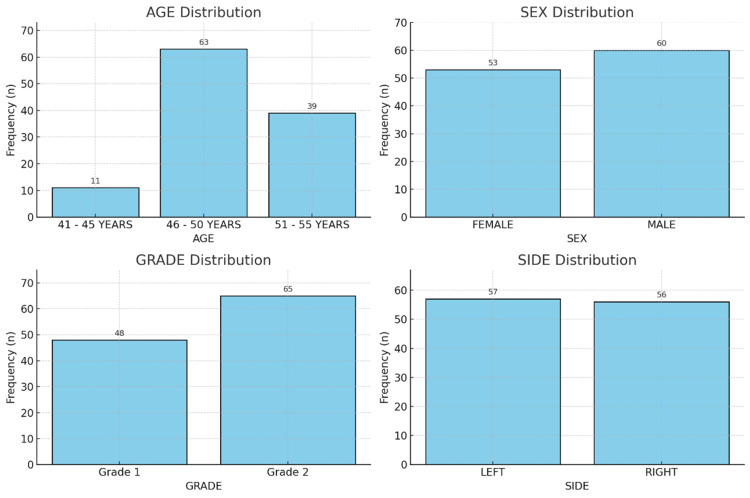
Demographic and clinical characteristics of study participants

The study involved 113 patients diagnosed with early-stage knee OA who had intra-articular PRP injections for 24 weeks to assess clinical and functional outcomes. The demographic information of the research population is summarized in Table 1 and Figure 2. Most patients (63, 55.8%) were in the 46-50 age range, followed by 39 (34.5%) in the 51-55 category, and 11 (9.7%) in the 41-45 group, indicating that knee OA predominantly affects individuals in their late 40s to early 50s. Among the participants, 60 (53.1%) were men and 53 (46.9%) were women. Based on Kellgren-Lawrence grading, 48 patients (42.5%) had grade 1 OA, while 65 (57.5%) had grade 2 OA. Lateral involvement was nearly equal, with 57 cases (50.4%) affecting the left knee and 56 (49.6%) the right. Overall, Table 1 and Figure 2 depict a middle-aged cohort with early-stage OA and a balanced distribution of sex and side of involvement, providing a representative sample for evaluating PRP treatment efficacy.

**Table 1:** Demographic and clinical characteristics of study participants

Variable	Category	Frequency	Percentage	Valid percentage
Age group, years	41-45	11	9.70%	9.70%
	46-50	63	55.80%	55.80%
	51-55	39	34.50%	34.50%
	Total	113	100.00%	100.00%
Gender	Female	53	46.90%	46.90%
	Male	60	53.10%	53.10%
	Total	113	100.00%	100.00%
Kellgren-Lawrence grade	Grade 1	48	42.50%	42.50%
	Grade 2	65	57.50%	57.50%
	Total	113	100.00%	100.00%
Side	Left	57	50.40%	50.40%
	Right	56	49.60%	49.60%
	Total	113	100.00%	100.00%

Table 2 compares functional (WOMAC) and pain (VAS) scores between patients with grade 1 and grade 2 OA at various time points. Across all follow-up periods (pre-injection, six weeks, 12 weeks, and 24 weeks), patients with grade 1 OA consistently exhibited lower WOMAC and VAS scores than those with grade 2 OA. At baseline, the mean WOMAC scores were 31.27 for grade 1 and 33.95 for grade 2, which improved to 23.9 and 25.63, respectively, by week 24. Similarly, VAS scores declined from 5.38 to 2.56 in grade 1 and from 6.23 to 3.06 in grade 2 over the same period. All differences were statistically significant (p < 0.001), indicating that while both groups experienced clinical improvement following PRP therapy, patients with milder disease demonstrated a more pronounced response. Thus, Table 2 highlights that PRP treatment effectively alleviates symptoms in both OA stages, with better outcomes observed in grade 1 patients.

**Table 2:** Comparison of WOMAC and VAS scores between grade 1 and grade 2 patients WOMAC: The Western Ontario and McMaster Universities Arthritis Index; VAS: visual analog scale; SD: standard deviation; SEM: standard error of the mean; CI: confidence interval

Score type	Time point	Grade	N	Mean	SD	SEM	95% CI lower	95% CI upper	P-value
WOMAC Index score	Pre-injection	Grade 1	48	31.27	1.842	0.266	-3.449	-1.917	0.00
		Grade 2	65	33.95	2.161	0.268	-3.431	-1.935	0.00
	6th week post-injection	Grade 1	48	29.23	2.126	0.307	-2.944	-1.459	0.00
		Grade 2	65	31.43	1.845	0.229	-2.962	-1.441	0.00
	12th week post-injection	Grade 1	48	27.48	1.762	0.254	-2.521	-1.259	0.00
		Grade 2	65	29.37	1.606	0.199	-2.531	-1.249	0.00
	24th week post-injection	Grade 1	48	23.9	1.547	0.223	-2.381	-1.089	0.00
		Grade 2	65	25.63	1.825	0.226	-2.365	-1.105	0.00
VAS score	Pre-injection	Grade 1	48	5.38	0.64	0.092	-1.101	-0.611	0.00
		Grade 2	65	6.23	0.656	0.081	-1.1	-0.612	0.00
	6th week post-injection	Grade 1	48	4.69	0.624	0.09	-0.831	-0.379	0.00
		Grade 2	65	5.29	0.579	0.072	-0.833	-0.376	0.00
	12th week post-injection	Grade 1	48	3.85	0.684	0.099	-0.674	-0.202	0.00
		Grade 2	65	4.29	0.579	0.072	-0.681	-0.196	0.00
	24th week post-injection	Grade 1	48	2.56	0.649	0.094	-0.724	-0.274	0.00
		Grade 2	65	3.06	0.556	0.069	-0.73	-0.268	0.00

Paired t-tests were used over time to assess changes in WOMAC scores, as presented in Table 3. At each follow-up interval, the mean baseline WOMAC score of 32.81 showed a steady and statistically significant reduction: to 30.50 at six weeks, 28.57 at 12 weeks, and 24.89 at 24 weeks. The most substantial improvement occurred at 24 weeks, with a mean decrease of more than 8 points from baseline (p < 0.001). These findings indicate continuous functional improvement over time, supporting the sustained efficacy of PRP in enhancing joint mobility and reducing stiffness. Overall, Table 3 demonstrates that PRP therapy led to consistent and significant functional gains over the six-month follow-up period.

**Table 3:** Paired T-test comparison of WOMAC scores at different time points ^*^Paired t-test WOMAC: The Western Ontario and McMaster Universities Arthritis Index; SD: standard deviation; SEM: standard error of the mean; CI: confidence interval

Pair	Time point	Mean	N	SD	SEM	Correlation (r)	Sig. (r)	95% CI lower	95% CI upper	P-value*
Pair 1	WOMAC pre-injection	32.81	113	2.422	0.228	0.878	0.00	2.101	2.536	0.00
	WOMAC 6TH week post-injection	30.5	113	2.245	0.211					
Pair 2	WOMAC pre-injection	32.81	113	2.422	0.228	0.773	0.00	3.961	4.535	0.00
	WOMAC 12TH week post-injection	28.57	113	1.913	0.18					
Pair 3	WOMAC pre-injection	32.81	113	2.422	0.228	0.573	0.00	7.538	8.303	0.00
	WOMAC 24TH week post-injection	24.89	113	1.91	0.18					

Table 4 demonstrates a comparable trend over time when assessing pain outcomes using VAS scores. The mean VAS score showed a statistically significant reduction at each follow-up (p < 0.001), decreasing from 5.87 at baseline to 5.04 at six weeks, 4.11 at 12 weeks, and 2.85 at 24 weeks. The most pronounced decline occurred between baseline and week 24, indicating a strong and sustained analgesic effect of PRP. These findings suggest that PRP not only provides early pain relief but also ensures lasting pain control. Thus, Table 4 concludes that the steady reduction in pain associated with early-stage OA can be effectively achieved through intra-articular PRP injections.

**Table 4:** Paired comparison of VAS scores at different time points ^*^Paired t-test VAS: visual analog scale; SD: standard deviation; SEM: standard error of the mean; CI: confidence interval

Pair	Time point	Mean	N	SD	SEM	Correlation (r)	Sig. (r)	95% CI lower	95% CI upper	P-value
Pair 1	VAS pre-injection	5.87	113	0.773	0.073	0.736	0.00	0.733	0.931	0.00
	VAS 6TH week post-injection	5.04	113	0.667	0.063					
Pair 2	VAS pre-injection	5.87	113	0.773	0.073	0.483	0.00	1.624	1.898	0.00
	VAS 12TH week post-injection	4.11	113	0.66	0.062					
Pair 3	VAS pre-injection	5.87	113	0.773	0.073	0.372	0.00	2.868	3.167	0.00
	VAS 24TH week post-injection	2.85	113	0.644	0.061					

Table 5 presents the Pearson correlation analysis between age, radiographic grade, and outcome measures. Across all time points -most notably at six weeks post-injection (r = 0.833)- age showed a strong positive correlation with both WOMAC and VAS scores, indicating that older patients tended to have poorer outcomes. Radiographic grade also demonstrated modest yet significant positive correlations with WOMAC and VAS scores, with higher grades associated with worse functional and pain scores. Additionally, WOMAC and VAS scores at corresponding time intervals were strongly correlated, confirming the reliability and consistency of the outcome assessments. Overall, Table 5 illustrates that clinical outcomes are influenced by both age and OA severity, with younger patients and those with milder disease deriving greater benefit from PRP therapy

**Table 5:** Pearson correlation matrix of age, grade, and WOMAC and VAS scores at sixth, 12th, and 24th week post--injection *All correlations are significant at the 0.01 level Pearson correlation values indicate the strength and direction of the linear relationship between the variables. N = 113 for all correlations WOMAC: The Western Ontario and McMaster Universities Arthritis Index; VAS: visual analog scale

Variable	Age	Grade	WOMAC 6th week post-injection	VAS 6th week post-injection	WOMAC 12th week post-injection	VAS 12th week post-injection	WOMAC 24th week post-injection	VAS 24th week post-injection
Age	1	0.581^*^	0.833^*^	0.626^*^	0.776^*^	0.590^*^	0.644^*^	0.519^*^
Grade	0.581^*^	1	0.487^*^	0.450^*^	0.491^*^	0.330^*^	0.451^*^	0.385^*^
WOMAC 6th week post-injection	0.833^*^	0.487^*^	1	0.710^*^	0.872^*^	0.555^*^	0.729^*^	0.596^*^
VAS 6th week post-injection	0.626^*^	0.450^*^	0.710^*^	1	0.600^*^	0.539^*^	0.570^*^	0.574^*^
WOMAC 12th week post-injection	0.776^*^	0.491^*^	0.872^*^	0.600^*^	1	0.653^*^	0.774^*^	0.679^*^
VAS 12th week post-injection	0.590^*^	0.330^*^	0.555^*^	0.539^*^	0.653^*^	1	0.604^*^	0.711^*^
WOMAC 24th week post-injection	0.644^*^	0.451^*^	0.729^*^	0.570^*^	0.774^*^	0.604^*^	1	0.720^*^
VAS 24th week post-injection	0.519^*^	0.385^*^	0.596^*^	0.574^*^	0.679^*^	0.711^*^	0.720^*^	1

Taken together, the findings clearly demonstrate that intra-articular PRP injections significantly enhance both pain relief and functional performance in patients with early-stage knee OA. Individuals with grade 1 disease and younger age experienced more pronounced and sustained improvements. Overall, these results support PRP as an effective and minimally invasive therapeutic option for managing early knee OA, with clinically meaningful benefits maintained over 24 weeks.

## Discussion

Over the 24-week follow-up period, we observed significant and continuous improvements in both pain relief and functional outcomes among patients with early-stage knee OA treated with intra-articular PRP injections. These findings highlight the therapeutic potential of PRP in OA management and align with the growing body of supportive evidence. The majority of our participants had Kellgren-Lawrence grade 1 or 2 OA and were aged 46-50 years, consistent with the demographic profiles typically studied in regenerative medicine research, thereby allowing meaningful comparison with previous trials.

A similar trend of statistically significant improvements in both VAS and WOMAC scores was observed when comparing our results with those of Moretti et al., who evaluated 153 patients receiving three PRP injections over six months. Like our study, their findings demonstrated notable clinical and functional improvements without corresponding radiographic changes in cartilage. This agreement supports the notion that PRP’s therapeutic benefits may primarily stem from its anti-inflammatory and pain-modulating effects rather than from immediate structural regeneration. Interestingly, while Moretti et al. employed a three-injection protocol, our study (using a single PRP injection) showed sustained benefits for up to 24 weeks. This suggests that a single injection could offer comparable mid-term clinical advantages in early OA patients, which is clinically relevant in terms of cost-effectiveness and patient compliance [12].

Chu et al. (2022) conducted a large-scale, multicenter randomized controlled trial over 60 months comparing pure PRP (P-PRP) with saline. Their study demonstrated consistently greater improvements in WOMAC and VAS scores in the PRP group across all follow-up intervals, along with a slower decline in cartilage volume. Notably, the most significant improvement in our study occurred at 24 weeks, corresponding to the six-month peak observed in Chu et al.’s findings. Both studies indicate that PRP provides durable symptomatic relief, particularly in reducing pain and enhancing physical function. Although our research did not include biomarker analysis, Chu et al. also reported favorable biochemical changes in inflammatory mediators such as TNF-α and IL-1β. Nevertheless, our clinical outcomes align closely with theirs - especially among grade 1 OA patients, where benefits were more pronounced -highlighting the need for early management [13].

Huang et al. (2022) conducted a randomized controlled trial examining the combined effect of two different hyaluronic acid formulations with PRP. While differences between groups were minimal and PRP remained the primary therapeutic agent, both combination treatments led to significant improvements in VAS and WOMAC scores after six months. Similarly, our study demonstrated substantial pain relief and functional gains with PRP alone, suggesting that supplementary HA may not be necessary in early OA cases. Furthermore, our results support the feasibility of PRP monotherapy, which streamlines treatment logistics without compromising clinical outcomes [14].

In contrast, Bennell et al. (2022) reported no significant changes in symptoms or joint structure at 12 months when comparing PRP with saline in a well-powered, placebo-controlled trial. However, their study included patients with Kellgren-Lawrence grade 2-3 OA, representing a somewhat more advanced population than our grade 1-2 cohort, which may explain the differences in observed efficacy. Our findings, along with those of Moretti et al. and Chu et al., support the notion that PRP is most effective in early-stage OA. Additionally, while Bennell et al. focused on radiologic outcomes and observed minimal structural change, our study prioritized patient-reported outcome measures (PROMs), which consistently demonstrated clinical improvement. This highlights the importance of selecting appropriate endpoints in PRP research, particularly when symptom relief is the primary goal [15].

Beiki et al. (2024) also reported findings supporting PRP, with notable improvements in WOMAC and International Knee Documentation Committee (IKDC) scores, although their comparison with a needling-only control group showed no significant intergroup differences in WOMAC or EQ-VAS at six months. Interestingly, their study demonstrated more pronounced gains in functional measures (Tegner Activity Score (TAS), IKDC), aligning with our results, which showed consistent improvements in WOMAC function subscales over 24 weeks. While their work emphasized the safety and feasibility of PRP, our study more clearly establishes its therapeutic efficacy, particularly in younger patients and those with grade 1 OA, who experienced the most favorable outcomes [16].

The significant associations observed in our data between older age, higher radiographic grade, and poorer VAS and WOMAC scores mirror previous research indicating that patient selection critically influences treatment outcomes. Our study showed superior results in younger patients and those with less severe radiographic OA, a pattern also noted by Chu et al. and Moretti et al., emphasizing the importance of early intervention to maximize PRP efficacy. Taken together, these findings substantially advance current understanding of PRP in OA management. Specifically, they provide strong evidence that a single intra-articular PRP injection can deliver meaningful and sustained improvements in pain and joint function over six months in early-stage OA patients. For individuals seeking alternatives to long-term pharmacologic therapy, our results support PRP as a well-tolerated, minimally invasive, and effective treatment option.

Limitations

This study has a few limitations, especially its Small sample size, limited generalizability, and comparative interpretation. The 24-week follow-up may not be sufficient to capture long-term results or disease-modifying effects. Moreover, no imaging or biochemical markers were used to objectively evaluate structural changes or inflammation.

Recommendations

Intra-articular PRP injection is recommended as a safe and effective treatment for early-stage (grade 1 and 2) knee OA, especially in middle-aged patients, as it offers substantial improvements in pain relief and functional outcomes. Clinicians should consider PRP therapy as a minimally invasive alternative for patients who do not respond to conventional treatments, particularly before contemplating surgical interventions.

## Conclusions

Our findings show that individuals with early-stage knee OA experience significantly improved pain and functional results after intra-articular PRP injection. Notable improvements in WOMAC and VAS scores were observed as early as six weeks and persisted through 24 weeks, especially in younger patients and those with grade 1 OA. These results confirm the clinical use of PRP as a safe, minimally invasive, and efficient therapeutic option for early knee OA. Further large-scale, long-term studies are needed to optimize treatment protocols and evaluate the potential disease-modifying effects of PRP therapy.
